# Advances in Clinical Imaging of Vascular Inflammation: A State-of-the-Art Review

**DOI:** 10.1016/j.jacbts.2023.10.007

**Published:** 2023-12-13

**Authors:** Henry W. West, Katerina Dangas, Charalambos Antoniades

**Affiliations:** a Acute Multidisciplinary Imaging and Interventional Centre, Radcliffe Department of Medicine, University of Oxford, Oxford, United Kingdom; b Central Clinical School, Sydney Medical School, University of Sydney, Sydney, New South Wales, Australia

**Keywords:** atherosclerosis, cardiac magnetic resonance, computed tomography, imaging, inflammation, positron emission tomography

## Abstract

Vascular inflammation is a major contributor to cardiovascular disease, particularly atherosclerotic disease, and early detection of vascular inflammation may be key to the ultimate reduction of residual cardiovascular morbidity and mortality. This review paper discusses the progress toward the clinical utility of noninvasive imaging techniques for assessing vascular inflammation, with a focus on coronary atherosclerosis. A discussion of multiple modalities is included: computed tomography (CT) imaging (the major focus of the review), cardiac magnetic resonance, ultrasound, and positron emission tomography imaging. The review covers recent progress in new technologies such as the novel CT biomarkers of coronary inflammation (eg, the perivascular fat attenuation index), new inflammation-specific tracers for positron emission tomography–CT imaging, and others. The strengths and limitations of each modality are explored, highlighting the potential for multi-modality imaging and the use of artificial intelligence image interpretation to improve both diagnostic and prognostic potential for common conditions such as coronary artery disease.

## Highlights

• Vascular inflammation is a druggable target involved in the development and rupture of atherosclerotic plaques. Noninvasive imaging methodologies that quantify vascular inflammation can help in risk stratification and guide treatments.
• Molecular imaging using PET/PET-CT/PET-MRI adds significantly to the understanding of disease pathogenesis and is considered the gold standard in visualizing inflammation noninvasively. However, the practical limitations of its clinical deployment limit its use in clinical practice.
• CT imaging allows high-resolution investigation of plaque structure, allowing detection of high-risk features. CT phenotyping of PVAT for the assessment of vascular inflammation allows detection of residual inflammatory risk. This method is used in clinical practice.
• CMR allows structural assessment of large vessels, but its limited spatial and temporal resolution restricts its use for assessment of coronary plaque or coronary inflammation.
• Ultrasound offers an attractive perspective for assessment of vascular inflammation at low cost and with no radiation, but current approaches do not offer an alternative to PET imaging– or CT imaging–based methods for assessment of coronary inflammation.

Vascular inflammation plays a critical role in the development and progression of various cardiovascular diseases, most importantly atherosclerotic coronary artery disease (CAD). Accurate noninvasive assessment of vascular inflammation has been a challenge for clinicians and researchers due to the limitations of traditional imaging modalities, with invasive assessment limited to surgical patients in highly controlled research settings. With the advent of more advanced imaging technologies such as multi-detector computed tomography (CT) scanners and molecular imaging using selective radiotracers in positron emission tomography (PET), it is now possible to directly assess vascular biology and inflammation, including in the coronary tree. Furthermore, the burgeoning field of CT radiomics has the potential to augment noninvasive inflammation detection for improvements in patient care. Concurrently, molecular imaging with PET has experienced tremendous growth in recent years, with numerous radiotracers and imaging technologies being developed for the detection of vascular inflammation. Intravascular ultrasound is also becoming increasingly available during invasive angiography, presenting a significant opportunity for the use of ultrasound-based methods for the indirect detection of the high-risk (and presumably inflamed) coronary plaque.

In the current state-of-the-art review, an overview is provided of the advances in all imaging technologies used for the detection of vascular inflammation, with a specific focus on coronary artery inflammation. The review focuses on CT imaging techniques, which have seen huge interest in recent years, but it also includes dedicated discussion of magnetic resonance imaging (MRI), PET-CT and PET-MRI, and ultrasound. Highlighted also is the recent progress in human translational applications of each imaging modality, evaluating their strengths and limitations in the assessment of vascular inflammation.

## Introduction to Vascular Inflammation

Atherosclerosis is a chronic inflammatory condition of the vasculature. It is highly patterned in its pathophysiology, forming characteristic lesions within the arterial system all around the body. Broadly, atherosclerosis encompasses the stages of endothelial dysfunction, formation of fatty streak, plaque development (atheroma and fibroatheroma), and plaque disruption and thrombosis, including the pathophysiological processes that underpin these transitions. The spectrum of disease caused by atherosclerosis is vast, including myocardial infarction (MI), stroke, and peripheral arterial disease, which, among other related conditions, comprise cardiovascular disease, the leading cause of morbidity and mortality globally.^1^

**Figure 1:**
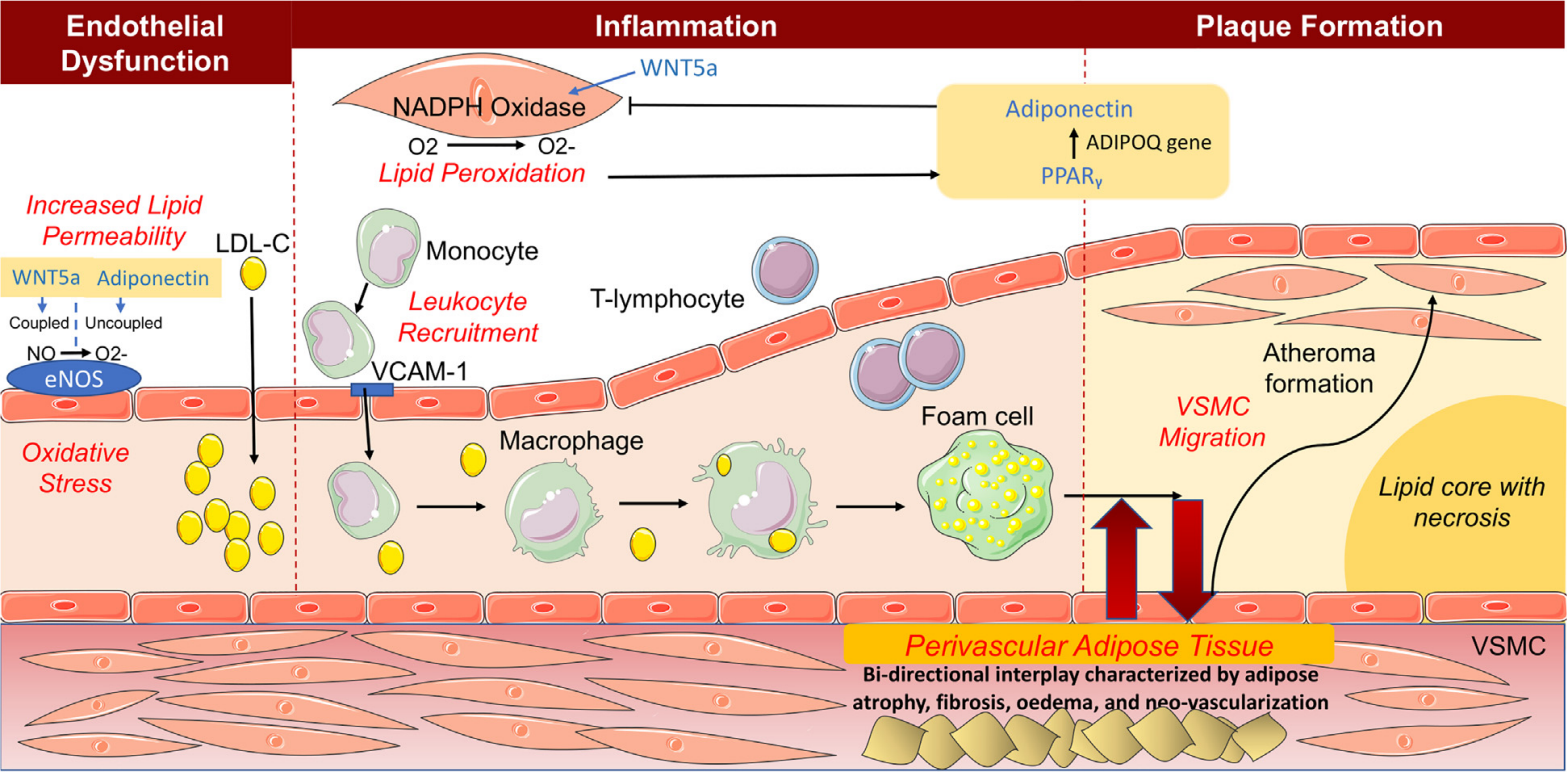
Simplified Schematic of Vascular Inflammation Driving Atherosclerosis Endothelial dysfunction results in collagen exposure and adhesion of circulating immune cells to the vascular wall at sites of up-regulation of adhesion markers such as intercellular adhesion molecule (ICAM)-1 and vascular cell adhesion molecule. The immune cells that migrate into the arterial wall along with circulating low-density lipoprotein cholesterol (LDL-C) initiate inflammatory responses in the subendothelial space. This mechanism drives the creation of foam cells and the organization of healing processes such as fibrotic change within the intima and the migration of smooth muscle cells into the atherosclerotic site. The influence of this process extends beyond the arterial intima and affects the arterial media and adventitia, including perivascular adipose tissue. eNOS = endothelial nitric oxide synthase; NADPH = nicotinamide adenine dinucleotide phosphate; NO = nitric oxide; O_2_ = oxygen; PPAR_γ_ = peroxisome proliferator-activated receptor-γ; VCAM-1 = vascular cell adhesion protein-1; VSMC = vascular smooth muscle cell.

Since the pioneering work of Russell Ross, vascular inflammation has played a causal role in the pathogenesis of all stages of atherosclerosis and plaque rupture.^2^, ^3^, ^4^ However, our understanding has flourished since Ross’s canonical response-to-injury hypothesis,^5^^,^^6^ which suggested that endothelial dysfunction (caused by genetics, vascular injury, elevated low-density lipoprotein [LDL-C] levels, free radicals from cigarette smoking, and hypertension, among others) causes collagen exposure and platelet adhesion, aggregation, and degranulation to initiate atherosclerosis. Adhesion markers (intercellular adhesion molecule-1, vascular cell adhesion molecule [VCAM-1]), in conjunction with chemotactic agents (chemokine [C-C motif] ligand 5) secreted by platelet degranulation, then act to stimulate neutrophil and macrophage migration and subendothelial accumulation of monocytes and LDL-C, initiating the inflammatory process.^7^ Other cytokines such as chemokine (C-C motif) ligand 2 are also released by neutrophils and smooth muscle cells (SMCs) and stimulate further leukocyte chemotaxis. The evolution of plaque then begins in the arterial intima, which contains a lipid-rich core from accumulation of LDL-C within macrophage foam cells underneath a fibrous cap formed from SMC migration and proliferation (Figure 1).

This canonical view of the initiation of atherosclerosis has been updated to reveal a complex interrelated network of pathways involving not only the innate immune system but also adaptive immunity (T- and B-cell responses), thrombo-inflammation (platelet-triggered processes), and C-mediated processes, among others. The role of B cells in atherosclerosis is complex,^8^ as some B-cell responses targeting oxidation-specific epitopes might be disease protective, whereas other downstream pathways may be pro-atherosclerotic. Furthermore, antibody-independent roles include cytokine production and T-cell regulation that secondarily mediate atherosclerosis. B-cell depletion therapies have been investigated, but further isolation of the pro-atherosclerotic pathways may be even more useful in generating targeted therapies. A role for T helper 1 cells is also being established.^9^ Platelets have been known to be critical in the early stages of atherosclerosis by releasing chemokine (C-C motif) ligand 5, a critical chemokine for promoting monocyte adhesion; increasingly complex roles are being elucidated.^10^ The role of these cell-mediated pathways and others (eg, protein kinase C) have been reviewed in detail elsewhere.^11^

The role of inflammation also extends to atherosclerotic disease progression.^7^ Activated macrophages that ingest lipid molecules subsequently secrete chemokines and cytokines and drive lipid accumulation.^12^^,^^13^ Notably, however, the role of macrophages in atherosclerosis is highly complex, with both pro- and anti-atherosclerotic functions. One recent study suggests that mural cell–driven macrophage niches may be protective against chronic inflammation.^14^ Furthermore, as SMCs and macrophages apoptose and a necrotic core grows, senescent SMCs release pro-inflammatory cytokines and matrix metalloproteinases.^15^ Oxidized LDL-C also triggers the nuclear factor κB signaling pathway, promoting the transcription of the NOD-, LRR-, and pyrin domain–containing 3 (NLRP3) inflammasome and pro–interleukin-1, triggering downstream pathways, which include the release of neutrophil extracellular traps. These traps further induce cytotoxicity by priming the NLRP3 inflammasome in macrophages and inducing platelet activation and the tissue factor pathway inhibitor.^7^

Finally, inflammation is also a critical driver of vulnerable plaque rupture, which is a key pathogenic mechanism of acute coronary syndromes (ACS). When the weakened cap breaks, procoagulant molecules in the blood are exposed to tissue factor within the lipid-rich core; platelet aggregation and thrombosis ensue. Superficial erosion is a second mechanism by which plaque progression occurs; depletion of neutrophils has been shown to prevent this process, further highlighting the role of inflammation.^16^

Vascular inflammation is therefore causally implicated in atherosclerosis from inception to complication. Clinical studies have concordantly confirmed the clinical relevance of inflammation in atherosclerotic cardiovascular disease. Elevation in inflammatory markers predicts outcomes in patients with ACS.^17^, ^18^, ^19^ Inflammation also affects cardiovascular risk factors, possibly by decreasing nitric oxide bioavailability in the vascular endothelium.^20^ Vascular inflammation has furthermore been shown to affect serum lipid levels and lipoprotein function. It causes LDL-C to be more easily oxidized,^21^ as the ability of HDL to prevent the oxidation of LDL-C is diminished. It follows that lipid-modifying statins trigger clinically significant anti-inflammatory processes.^22^

Therefore, targeting these complex pathways provides a promising route toward diagnosing and treating the disease. Our growing understanding of the complex interconnected pathways that give rise to the modern view of atherosclerosis serves as a potential avenue for precision medicine. Inflammation had been largely overlooked until recent years but has become a fast-growing target for innovation in diagnostics and treatment. In particular, the potential to further stratify CAD according to the presence of inflammation may be critical, as anti-inflammatory treatments for atherosclerosis are recommended in clinical guidelines.^23^ Its potential applications are also being investigated beyond ischemic heart disease to include cardiometabolic disease.^24^

## Causal Relationships Between Inflammation and Cardiovascular Events: Insights From Randomized Clinical Trials

The advance in understanding of the cellular mechanisms of the chronic inflammatory process underlying atherosclerosis is increasingly being translated into actionable clinical discoveries. Phase 3, double-blind, randomized controlled clinical trials have now shown that targeting specific inflammatory pathways can improve clinical outcomes and reduce cardiovascular events in select populations. Importantly, different anti-inflammatory strategies may have varying agent-related effectiveness due to diversity in underlying mechanisms, specifically whether they prevent early disease progression, lessen plaque formation, or reduce late-stage plaque rupture to prevent acute cardiac events. This scenario highlights a major challenge in clinical trials for anti-inflammatory agents: what is the population of relevance for the specific agent, and what is the relevant outcome that the agent may modify?

The first trial to test a clinical treatment strategy based on inflammatory markers was JUPITER (Justification for the Use of Statin in Prevention: An Intervention Trial Evaluating Rosuvastatin). In 17,802 patients selected per LDL-C level <130 mg/dL and high-sensitivity C-reactive protein level ≥2 mg/L, rosuvastatin 20 mg was associated with a reduction in the rate of primary endpoint (MI, stroke, arterial revascularization, hospitalization for unstable angina, and cardiovascular death) compared with placebo (rosuvastatin vs placebo 1.31 vs 0.77).^25^ However, it was unclear whether it was an on-target effect due to LDL-C reduction or a pleiotropic effect of reducing inflammation, generating the need for investigation and development of targeted anti-inflammatory treatments.

Notably, CANTOS (Canakinumab Anti-inflammatory Thrombosis Outcomes Study) investigated the impact of an interleukin-1β–targeted monoclonal antibody (canakinumab) on cardiovascular outcomes, in 10,061 patients with a previous MI and C-reactive protein level ≥2 mg/L.^26^ The trial compared 3 doses (50, 150, and 300 mg) of canakinumab administered subcutaneously every 3 months vs placebo on the primary endpoint of nonfatal MI, nonfatal stroke, or cardiovascular death. Canakinumab 150 mg every 3 months was found to significantly reduce recurrent major adverse cardiovascular events (MACE) by 17% (HR: 0.83; *P* = 0.005). This study was the first to show that anti-inflammatory drug treatment reduces cardiovascular risk, although the relatively modest reduction in MACE, with no significant effect on cardiac mortality, questioned the use of high-sensitivity C-reactive protein for patient selection; this introduced the need for more precise and sophisticated methods to identify those patients with high coronary inflammation who would benefit the most from targeted treatment. The CANTOS trial has revived the need to develop imaging biomarkers of coronary inflammation for use as a companion or complementary diagnostic tool.^27^

However, the story is not that simple. CIRT (Cardiovascular Inflammation Reduction Trial) investigated low-dose methotrexate vs placebo in 4,786 patients with previous MI or multivessel coronary disease with diabetes or metabolic syndrome; it found no reduction in the composite primary endpoint (nonfatal MI, nonfatal stroke, or cardiovascular death) with methotrexate.^28^ An ancillary study found that impaired coronary flow reserve was independently associated with increased inflammation and myocardial strain, which may have implications in heart failure.^24^ The contrasting results of the CIRT and CANTOS trials show that anti-inflammatory therapies effective against atherosclerosis must be biochemically targeted to the specific inflammatory pathways validated in the disease. Whereas the interleukin-1β to NLRP3 inflammasome pathway targeted by canakinumab is genetically and cellularly validated as a critical driver of atherosclerosis, this is not the case for nucleic acid synthesis targeted by methotrexate. Similarly, the phospholipase inhibitor darapladib^29^ and p38 mitogen-activated protein kinase blocker losmapimod^30^ also yielded neutral results as they act on pathways not likely critical in the pathophysiology of atherosclerosis. Thus, additional cellular work may be a critical hypothesis generator for future drug development.

Colchicine is a potent anti-inflammatory drug targeting cyclooxygenase-2 with wide-ranging clinical utility, including in gout and pericarditis. It is an effective tubulin disruptor and also a known inhibitor of leukocyte migration and of the NLRP3 inflammasome. This drug has shown promise in mitigating vascular inflammation in 2 key trials. COLCOT (Colchicine Cardiovascular Outcomes Trial) found that 0.5 mg daily colchicine vs placebo in patients <30 days after a MI significantly reduced the risk of ischemic cardiovascular events (cardiovascular death, resuscitated cardiac arrest, MI, stroke, and urgent hospitalization for angina leading to coronary revascularization) with colchicine than placebo.^31^ The effects of colchicine also translate to patients with chronic coronary disease, as shown by the LoDoCo2 (Low-Dose Colchicine 2) trial,^32^ which compared 0.5 mg colchicine daily vs placebo in those with chronic coronary disease. These 2 trials confirmed that anti-inflammatory treatments can be used in cardiovascular risk management and led to the inclusion of colchicine in the European Society of Cardiology 2021 prevention guidelines (with a Class IIb indication) as a therapeutic option on top of statins, in patients at very high risk.^33^

Furthermore, REDUCE-IT (Reduction of Cardiovascular Events with Icosapent Ethyl–Intervention Trial), which assessed icosapent ethyl in patients with hypertriglyceridemia, found a striking risk reduction of ischemic heart disease; however, the result was not found to be related to reduction in triglyceride level.^34^ It is indeed possible that this effect is mediated through the anti-inflammatory effects of this agent.

Finally, the ZEUS (Effects of Ziltivekimab vs Placebo on Cardiovascular Outcomes in Participants With Established Atherosclerotic Cardiovascular Disease, Chronic Kidney Disease, and Systemic Inflammation) trial investigating the novel interleukin-6 inhibitor ziltivekimab vs placebo on primary outcomes of cardiovascular death, nonfatal MI, and nonfatal stroke is ongoing.^35^ The results of this phase 3 trial have the potential to further shift the view of atherosclerosis from a lipid storage disease to an inflammatory one.

Overall, clinical practice changes with phase 3 randomized clinical trials. The most recent European Society of Cardiology guidelines reflect this paradigm shift by recommending colchicine in high-risk individuals with atherosclerotic cardiovascular disease.^33^ However, the direction of anti-inflammatory treatments to individuals with pathology that is amenable to such therapy is paramount. The side effect profiles of many anti-inflammatory treatments are not to be neglected, including infection risk and gastrointestinal complication resulting in poor tolerability. Furthermore, biologics are expensive therapies that require increased selectivity, which can be accomplished by personalized medicine. Further work is needed to develop reliable investigations to accurately identify those patients with arterial inflammation who would respond to such therapies. Emerging imaging techniques that allow measurement of cardiovascular inflammation are expected to soon lead us to more personalized therapeutic strategies.

## Noninvasive Detection of Vascular Inflammation

Noninvasive imaging techniques for the assessment of vascular inflammation have garnered significant interest in the research and clinical communities over recent years. As our understanding of the fundamental roles of inflammation in atherosclerosis and other vascular conditions such as vasculitis has improved, so has the impetus to use noninvasive means to assess human inflammatory load, particularly in the coronary arteries and the aorta. The noninvasive assessment of coronary artery inflammation has already been shown to provide significant opportunity for enhanced CAD risk stratification, personalized therapy decision-making, and enhanced monitoring of therapeutic efficacy^36^ (discussed in this section of the review).

We provide a review of the major noninvasive imaging modalities available to researchers and clinicians to visualize vascular inflammation in vivo with a focus on both the molecular mechanisms that these imaging modalities rely upon and their clinical applications. Invasive modalities such as intravascular imaging techniques are not included in this review. The noninvasive imaging modalities for the detection of vascular inflammation discussed here are summarized in the Central Illustration along with their strengths and weaknesses.

**Central Illustration:**
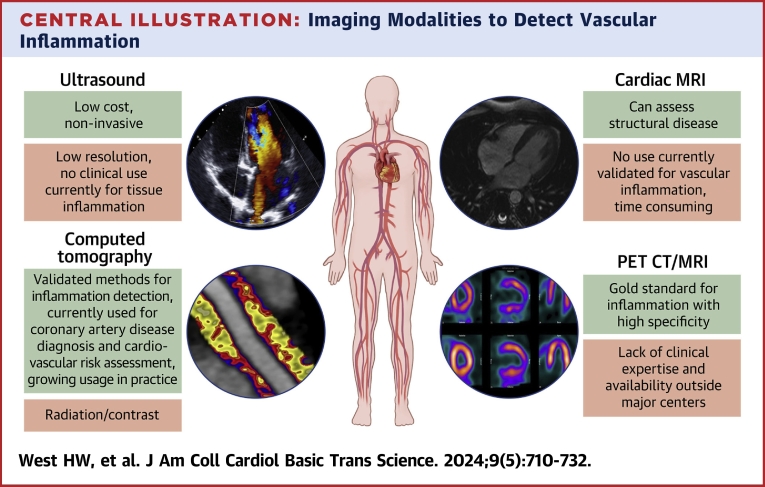
Imaging Modalities to Detect Vascular Inflammation Summary of the clinical imaging modalities in use or under development for the noninvasive detection of vascular inflammation. CT = computed tomography; MRI = magnetic resonance imaging; PET = positron emission tomography.

### CT Imaging

The use of CT imaging to visualize the heart and vascular structures has increased dramatically in recent decades owing to improved imaging technology and widespread utility of CT scanners across a broad range of clinical indications.

Coronary computed tomography angiography (CTA) has become the noninvasive imaging modality of choice for the noninvasive examination of the coronary arteries. The currently accepted broad indications for coronary CTA in clinical practice that are relevant to inflammatory vascular disease include: 1) suspected or known CAD (to evaluate the severity and extent of CAD, particularly in patients with symptoms such as stable chest pain, shortness of breath, and exercise intolerance); 2) assessing the anatomy of the coronary arteries before revascularization procedures such as coronary artery bypass surgery or percutaneous coronary intervention; 3) preoperative evaluation for noncardiac surgery to assess the presence and severity of CAD in patients scheduled for noncardiac surgery; and 4) monitoring the progression of CAD or assessing the effectiveness of treatment, including lifestyle changes, medication, or revascularization procedures.

It is important to note that coronary CTA is not always the first-line imaging modality for all these broad indications in all settings. The choice of imaging test often depends on an individual patient’s disposition.

Beyond the indications listed here, the role of CT imaging has recently expanded to include the direct noninvasive imaging of vascular inflammation. CT imaging offers unparalleled potential for widespread clinical uptake of vascular inflammation assessment, upheld by the existing reliance on CT scans in clinical guidelines for the investigation of chest pain worldwide.^37^^,^^38^ Advances that allow these scans to be further utilized for the visualization of coronary inflammation would improve and streamline clinical practice and add immense value for patients and clinicians.

Currently, when plaque is visualized with coronary CTA, plaque risk is stratified to assess plaque stability and therefore risk of cardiac events. High-risk plaque (HRP) features include low attenuation, positive remodeling, spotty calcification, and the napkin ring sign (ringlike peripheral higher attenuation with central low attenuation), among others. On the other hand, calcification signifies stability and low inflammation. However, HRP features are not reliable in assessing plaque inflammation. The absence of these features does not necessarily correlate with lack of inflammation because by the time plaque appears, vascular inflammation has been ongoing for some time. Indeed, recent studies show that the traditional stenosis-based approach has failed to identify at-risk patients (Coronary Artery Disease Reporting and Data System 2.0 [CAD-RADS 2.0])^39^ and furthermore failed to improve clinical outcomes beyond symptom improvement in patients with stable CAD (ISCHEMIA [International Study of Comparative Health Effectiveness With Medical and Invasive Approaches]).^40^ Therefore, to maximize prevention of cardiac events, imaging biomarkers specific to vascular inflammation are needed to detect coronary inflammation before plaque formation is detectable.

Perivascular adipose tissue (PVAT) can detect signals of disease emanating from the vascular wall. In brief, in conditions of cardiovascular disease, the arterial wall releases various mediators such as oxidation products (eg, 4-hydroxynonenal), which diffuse to PVAT, inducing the transformation of adipocytes from quiescent lipid-storage cells to active biosynthetic cells that secrete antioxidant adipokines such as adiponectin. These adipokines are then transported back to the vascular wall, acting as a defense mechanism against vascular oxidative damage. Inflammatory molecules originating from the vascular wall also diffuse into adjacent adipose tissue, preventing pre-adipocyte differentiation into mature adipocytes within PVAT. In addition, these inflammatory molecules stimulate perivascular lipolysis, generating a gradient of adipocyte size surrounding the inflamed artery. The adipocyte size gradient in PVAT close to the inflamed artery results in a higher lipid/water ratio in the layers of PVAT adjacent to the inflamed vascular wall. The gradient changes in PVAT’s structure and composition around inflamed arteries could act as an internal “thermometer” of vascular inflammation if it can be visualized and quantified noninvasively.

One of the key advances that has facilitated CT imaging to becoming a “one-stop-shop”^41^ for imaging of the vasculature, particularly the coronary arteries, is the fundamental but often overlooked fact that three-dimensional medical images, like all images, are data sets.

The first major imaging technology that utilizes coronary CTA for the detection of vascular inflammation is the perivascular fat attenuation index (FAI). This noninvasive, CT imaging–derived biomarker relies on attenuation mapping of pericoronary adipose tissue (PCAT) composition to extract information about the inflammatory status of the adjacent coronary artery.^42^^,^^43^ The premise of this work emerges from the understanding that adipose tissue is a key regulator of cardiometabolic health.^44^^,^^45^ PVAT is the adipose tissue that forms a contiguous entity with the arterial adventitia and plays a key role in vascular homeostasis and atherogenesis by regulating the local microenvironment through the release of bioactive adipokines,^46^^,^^47^ as well as gaseous and other lipid messengers.^42^^,^^44^

Studies that used ^18^F-fluorodeoxyglucose (^18^F-FDG) PET-CT imaging to visualize inflammation in PVAT (as outlined later in this review) have highlighted significant relationships between this inflammation and a range of clinically significant cardiovascular disease endpoints.^48^ Importantly, our group showed that the paracrine interactions between the arterial wall and the PVAT are bidirectional.^43^^,^^46^^,^^47^^,^^49^ We found that in the presence of increased vascular oxidative stress, lipid peroxidation products such as 4-hydroxynonenal are increasingly produced and diffuse from the vascular wall to the PVAT.^50^ These substances activate peroxisome proliferator-activated receptor-γ signaling in PVAT adipocytes, which results in an up-regulation and increased secretion of the antioxidant adiponectin from the perivascular adipocytes.^51^ Adiponectin can then diffuse back to the vascular wall and proximal myocardial tissue and reduce superoxide production by suppressing the activity of nicotinamide adenine dinucleotide phosphate oxidases, as well as by improving the coupling of endothelial nitric oxide synthase in the vascular endothelium.^46^^,^^47^^,^^52^ During the process of shifting the phenotype of PVAT adipocytes from energy storing to active secretory cells, their dimensions, shape, and content change, becoming smaller in size and with reduced intracellular lipid content.

The ability to evolve in response to signals from the cardiovascular system is also shown by the ability of adipocytes to activate lipolysis and reduce adipogenesis in the presence of exogenous inflammation and circulating molecules such as brain natriuretic peptide.^49^ Importantly, we have also shown that if vascular inflammation is present, the release of pro-inflammatory mediators such as tumor necrosis factor-α, interleukin-6, and interferon gamma blocks the ability of perivascular pre-adipocytes to differentiate into mature lipid-laden adipocytes.^43^ Indeed, per results of paired PVAT biopsies from a site attached to the right coronary artery, perivascular adipocytes were significantly smaller and less well differentiated compared with adipocytes from epicardial adipose tissue biopsies obtained >2 cm away from any coronary artery (non-PVAT); this was evidenced by a lower relative expression of the adipocyte differentiation markers peroxisome proliferator-activated receptor–γ and fatty acid binding protein-4. This gradient in PVAT composition reflects the inflammatory burden of a given coronary segment and has highlighted PVAT as a biological sensor of coronary artery inflammation. If these gradients of PVAT composition around the coronary arteries is visualized and quantified using noninvasive imaging, we would be able to detect or even quantify coronary artery inflammation noninvasively, leading to a new generation of diagnostic and prognostic biomarkers of cardiovascular events.

**Figure 2:**
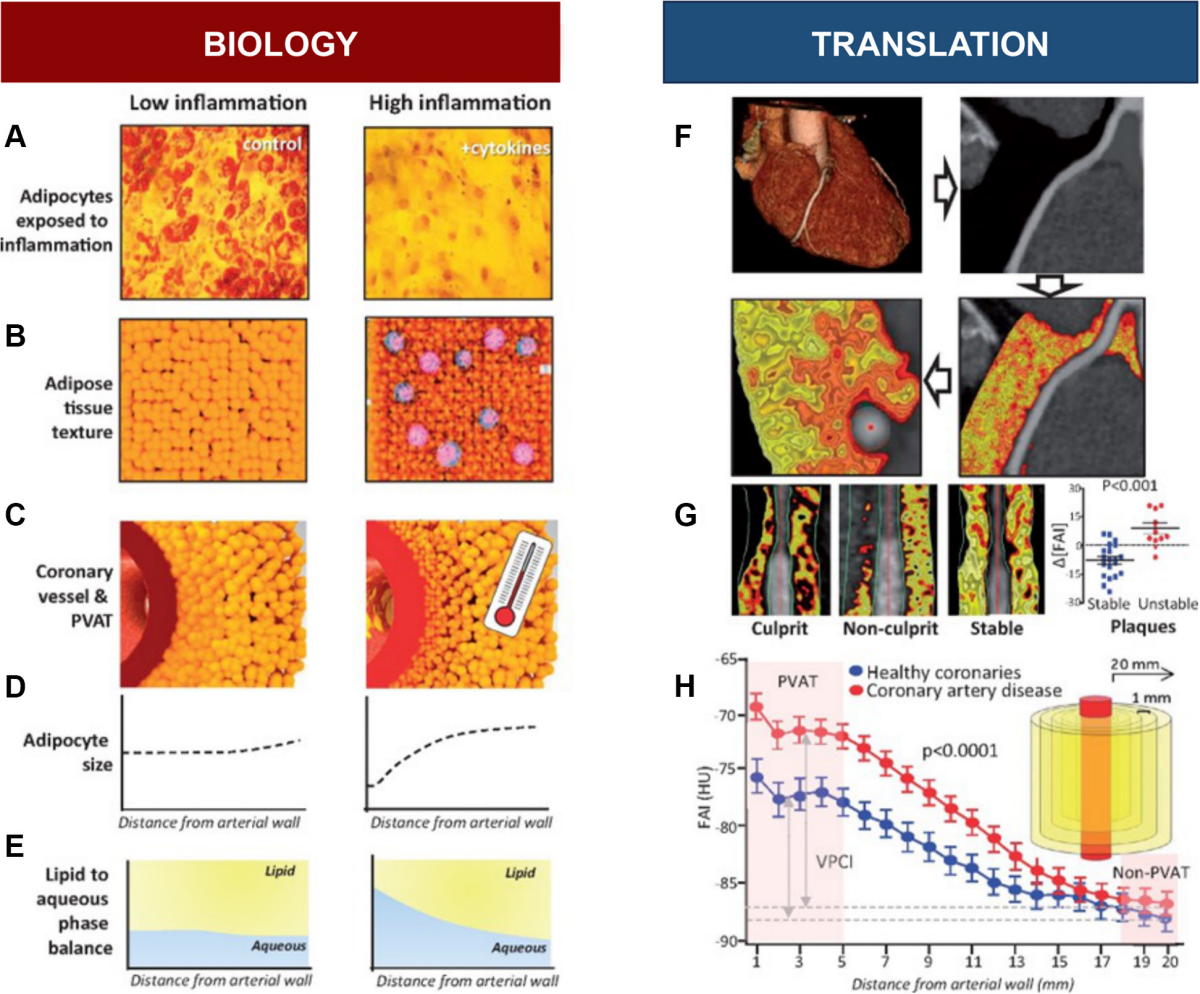
Schematic Representation of the Biology Underlying the Detection of Coronary Inflammation by Imaging PVAT (A) Exposure of adipose tissue to exogenous inflammation leads to smaller adipocytes and low levels of intracellular lipids. (B) Illustration of inflamed adipose tissue containing small adipocytes, with low intracellular fat levels and high macrophage infiltration. (C) Morphologic appearance of perivascular adipose tissue (PVAT) surrounding inflamed coronary arteries. Vascular inflammation leads to a gradient in the adipocyte size (D) and the lipid:aqueous phase in perivascular adipose tissue (E), which can be detected by coronary computed tomography angiography. (F) Standard-of-care routine coronary computed tomography angiography data sets can be used for fat attenuation index (FAI) measurements. Perivascular FAI mapping can discriminate unstable (ruptured) from stable atherosclerotic plaques (G) and is increased in patients with atherosclerosis (H). Reproduced with permission from Antoniades et al.^111^

These laboratory findings have been translated to coronary CTA through the segmentation and analysis of PVAT along the coronary vessels using predefined validated Hounsfield unit (HU) cutoffs (–190 to –30 HU).^43^^,^^53^ The perivascular FAI utilizes coronary CTA to track spatial changes in PVAT composition that are induced by inflamed coronary vessels as outlined earlier.^43^ The FAI relies on the concept that the inflammation-induced changes in adipocyte size are associated with a detectable shift in CT attenuation toward a less negative HU range (toward –30 HU). The perivascular FAI (calculated by using the CaRi-HEART medical device; Caristo Diagnostics^54^) captures and interprets these attenuation gradients in the perivascular space, with high perivascular FAI linked to a higher inflammatory burden^43^^,^^55^ (Figures 2A to 2E).

Importantly, there is strong evidence that CAD is associated with a higher perivascular FAI compared with healthy individuals.^43^ In addition, perivascular FAI is significantly increased around culprit/unstable lesions in patients presenting with acute MI, and perivascular FAI exhibits dynamic changes around culprit coronary lesions, decreasing significantly when measured 5 weeks after the index event. It is interesting to consider the concept of the vulnerable arterial plaque in light of these findings, as this has dominated clinical thinking around ACS for decades. It will be of tremendous importance whether inflammatory imaging, such as FAI, can assist in identifying coronary lesions at increased risk of rupture or, perhaps more importantly, superficial erosion.^56^ Whether CT imaging will be useful in noninvasive differentiation of ACS etiology (ie, plaque erosion vs rupture) remains to be seen. However, it is important to note that perivascular FAI provides a measure of vascular inflammation and CAD risk regardless of the presence of any detectable coronary plaque, suggesting that patient vulnerability extends beyond immediately detectable plaque(s). This has been confirmed by others,^56^^,^^57^ including a recent study of 765 coronary lesions by Kuneman et al,^58^ which found that mean unadjusted PCAT attenuation is significantly increased across culprit lesion precursors compared with non-culprit lesions in patients with ACS and vs lesions of patients with stable CAD, suggesting a higher intensity of inflammation.

Perivascular FAI has also been shown to accurately predict plaque progression^41^ and discriminate stable vs unstable atherosclerotic plaques in 2 independent cohorts (Figures 2F to 2H). These findings validate the utility of FAI for the detection of both global coronary artery inflammatory burden and clinically relevant plaques.

**Figure 3:**
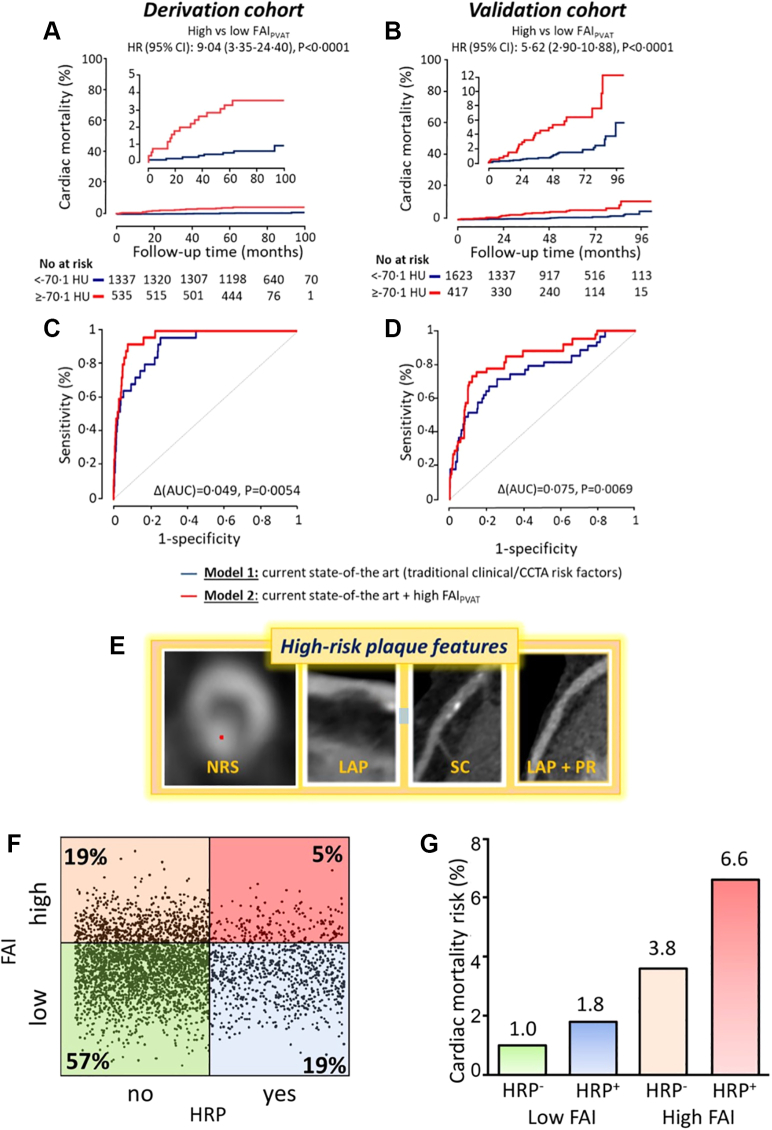
Prognostic Value of Perivascular FAI Prognostic value of perivascular FAI. (A, B) In the CRISP-CT (Cardiovascular Risk Prediction Using Computed Tomography) study, which evaluated 2 prospective clinical cohorts of 3,912 patients undergoing diagnostic coronary computed tomography angiography for clinical indications, perivascular FAI was predictive of cardiac mortality in both the derivation cohort and the validation cohort. (C, D) The FAI provided incremental prognostic value for cardiac mortality on top of traditional clinical risk factors, the Duke CAD Index, and number of high-risk plaque (HRP) features on coronary computed tomography angiography. Reproduced with permission from Oikonomou EK, Marwan M, Desai MY, et al. Non-invasive detection of coronary inflammation using computed tomography and prediction of residual cardiovascular risk (the CRISP CT study): a post-hoc analysis of prospective outcome data. *Lancet.* 2018;392:929-939. (E) HRP features on coronary computed tomography angiography are defined as the napkin-ring sign (NRS), low attenuation plaque (LAP), spotty calcification (SC), and positive remodeling (PR). (F, G) Stratification of the pooled population of CRISP-CT based on the presence of HRP and high coronary inflammatory burden as determined by the perivascular FAI and observed rates of cardiac mortality within each group. The combination of HRP and high FAI could be used to identify vulnerable patients at the highest risk who are eligible for aggressive prevention strategies; derived from post hoc data analysis of CRISP-CT data in the Oxford Academic Cardiovascular Computed Tomography Core Laboratory. Reproduced with permission from Antoniades et al.^111^ AUC = area under the curve; other abbreviations as in Figure 2.

The utility of FAI for cardiac mortality risk prediction has also been explored. Stratifying by high vs low FAI is shown to be able to predict risk of cardiac mortality (Figures 3A to 3D), with high FAI being associated with higher risk. This prognostic value was even incremental to current clinical risk factors (Duke CAD Index and HRP features on coronary CTA).

Furthermore, HRP features on coronary CTA in conjunction with FAI show promise as a powerful clinically relevant merged analysis for CAD risk stratification (Figures 3E to 3G). A recent narrative review suggests that a technology to improve plaque risk evaluation with coronary CTA would be of great use.^59^ Such findings have been confirmed by other studies, including the landmark SCOT-HEART (Scottish Computed Tomography of the Heart) trial,^60^ in which PCAT attenuation of the right coronary artery was predictive of MI events (HR: 1.55; 95% CI: 1.08-2.22; *P* = 0.017, per 1 SD increment). In multivariable analysis, adding PCAT of the right coronary artery −70.5 HU or higher to model including low-attenuation plaque burden led to improved prediction of future MI (HR: 11.7; 95% CI: 3.3-40.9; *P* < 0.0001). In a 2022 meta-analysis of 9 studies by Sagris et al,^61^ the power of FAI to identify stable and unstable coronary plaques (via the mean difference in attenuation) was tested. FAI was found to be significantly higher in unstable plaques compared with stable plaques with a mean difference of 4.5 HU (95% CI: 1.10-7.89; *I*^2^ = 88%). Higher pericoronary FAI values offered incremental prognostic value for MACE in studies with prospective follow-up (HR: 3.29; 95% CI: 1.88-5.76; *I*^2^ = 75%) among 6,335 patients.

Other groups have investigated the application of FAI to confirm the hypothesis behind the biomarker.^57^^,^^62^ In a validation of the link between coronary artery inflammation and PVAT phenotype, higher perivascular fat radiodensity has been shown to strongly correlate with both increased plaque inflammation as assessed by ^18^F-sodium fluoride (^18^F-NaF) uptake on PET-CT imaging^63^ and the progression of total and noncalcified atherosclerotic plaque burden in the adjacent vessel.^64^ It is noteworthy that in symptomatic patients undergoing cardiac CT imaging, the information captured by perivascular FAI is independent of coronary calcification^42^^,^^43^ or systemic markers of inflammation such as high-sensitivity C-reactive protein.^42^

Measuring perivascular attenuation in clinical practice is problematic, however, as it is affected by factors such as technical scan acquisition settings, image postprocessing, and local anatomical and biological factors.^65^ This has led to the development of a fully corrected metric of coronary inflammation, the FAI Score, which allows us to individualize the inflammatory burden of each coronary artery relative to the patient and use it as a clinical biomarker of coronary inflammation.^65^ Indeed, the FAI Score has received regulatory clearance (CE Mark) for use in clinical practice in the United Kingdom, Europe, and Australia, as the clinical metric of coronary inflammation obtained from coronary CTA. The medical device that measures the FAI Score (CaRi-HEART) incorporates these measurements and patient risk factors into a prognostic model of coronary atherosclerosis to generate the individualized patient risk for heart attack over a fixed period (using the CaRi-HEART risk calculator). The performance and prognostic value of the FAI Score in predicting cardiac mortality have been validated within the 2 original validation cohorts (Erlangen, n = 1,872; Cleveland Clinic, n = 2,040).^65^

**Figure 4:**
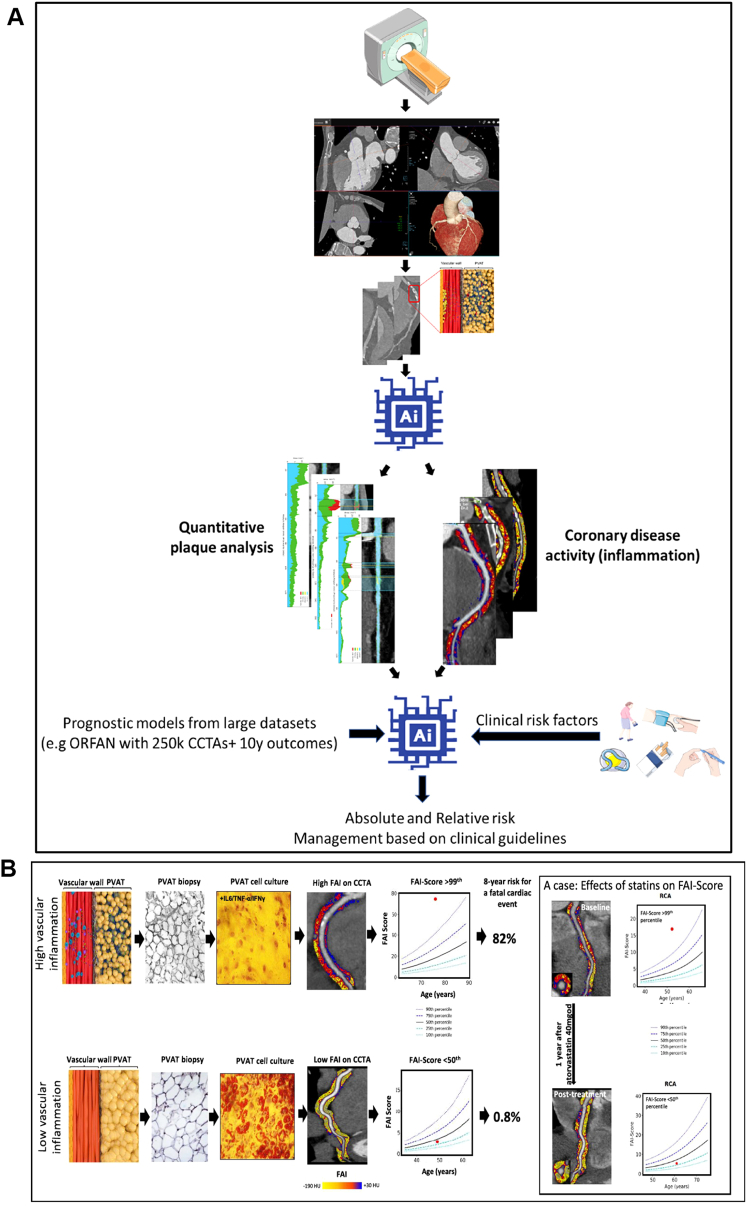
Concepts for Implementing Coronary Artery Plaque and Inflammation-Guided Management via CT Imaging in Clinical Practice (A) Example of workflow for artificial intelligence (Ai)-assisted computed tomography (CT) interpretation for the assessment of coronary artery disease, which includes automated prediction of a patient’s risk of major adverse cardiovascular events and suggested medical management. (B) Schematic representation of the biology underlying the detection of coronary inflammation by imaging PVAT. Health coronary artery shown on bottom and high inflammation at top, with corresponding low and high FAI Scores, respectively. At right is an example of a single patient with a high FAI Score at baseline with reduced vascular inflammation after 1 year of treatment with atorvastatin 40 mg once daily. A is reproduced with permission from Antoniades et al.^67^ B is reproduced with permission from Antoniades C et al.^111^ CCTA = coronary computed tomography angiography; ORFAN = Oxford Risk Factors and Non Invasive Imaging Study; RCA = right coronary artery; other abbreviations as in Figures 2 and 3.

Pericoronary FAI has been shown to be responsive to established CAD treatments, including 3-hydroxy-3-methylglutaryl coenzyme A reductase inhibitors (ie, statins). In a retrospective analysis of 108 patients who underwent coronary CTA,^66^ FAI was significantly lower in noncalcified plaques and mixed plaques when statin therapy was commenced (−68.0 ± 8.5 HU vs −71.5 ± 8.1 HU [*P* < 0.001] and −70.5 ± 8.9 HU vs −72.8 ± 9.0 HU [*P* = 0.014], respectively). This indicates potential for perivascular FAI as an imaging biomarker to monitor statin response. Indeed, Figure 4B presents a single case in which FAI was used to monitor the therapeutic response to statins through inflammatory burden in the right coronary artery. Currently, there is no direct noninvasive imaging test that clinicians can use to assess for potential response to such treatments.

The precise role for the perivascular FAI Score for assessment of coronary artery inflammation in clinical practice is in progress, with promising workstreams now being developed. Figure 4A presents the proposed workstream for incorporating inflammatory imaging into clinical CT imaging with automated artificial intelligence (AI) performing both quantitative plaque analysis and inflammatory imaging with FAI. The associated personalized risk would then be calculated when clinical risk factors are entered by clinicians,^67^ as PVAT inflammatory imaging is most powerful when integrated with plaque metrics and clinical risk factors (Figure 4B).

A recent clinical consensus statement from the European Society of Cardiology Working Group on Coronary Pathophysiology and Micro-circulation outlined current and future clinical applicability of AI technologies that integrate PVAT information into prognostic models.^68^ The goal is to provide clinically meaningful information in primary and secondary prevention of atherosclerotic heart disease, which offers the best high-level guidance for clinicians yet.

**Figure 5:**
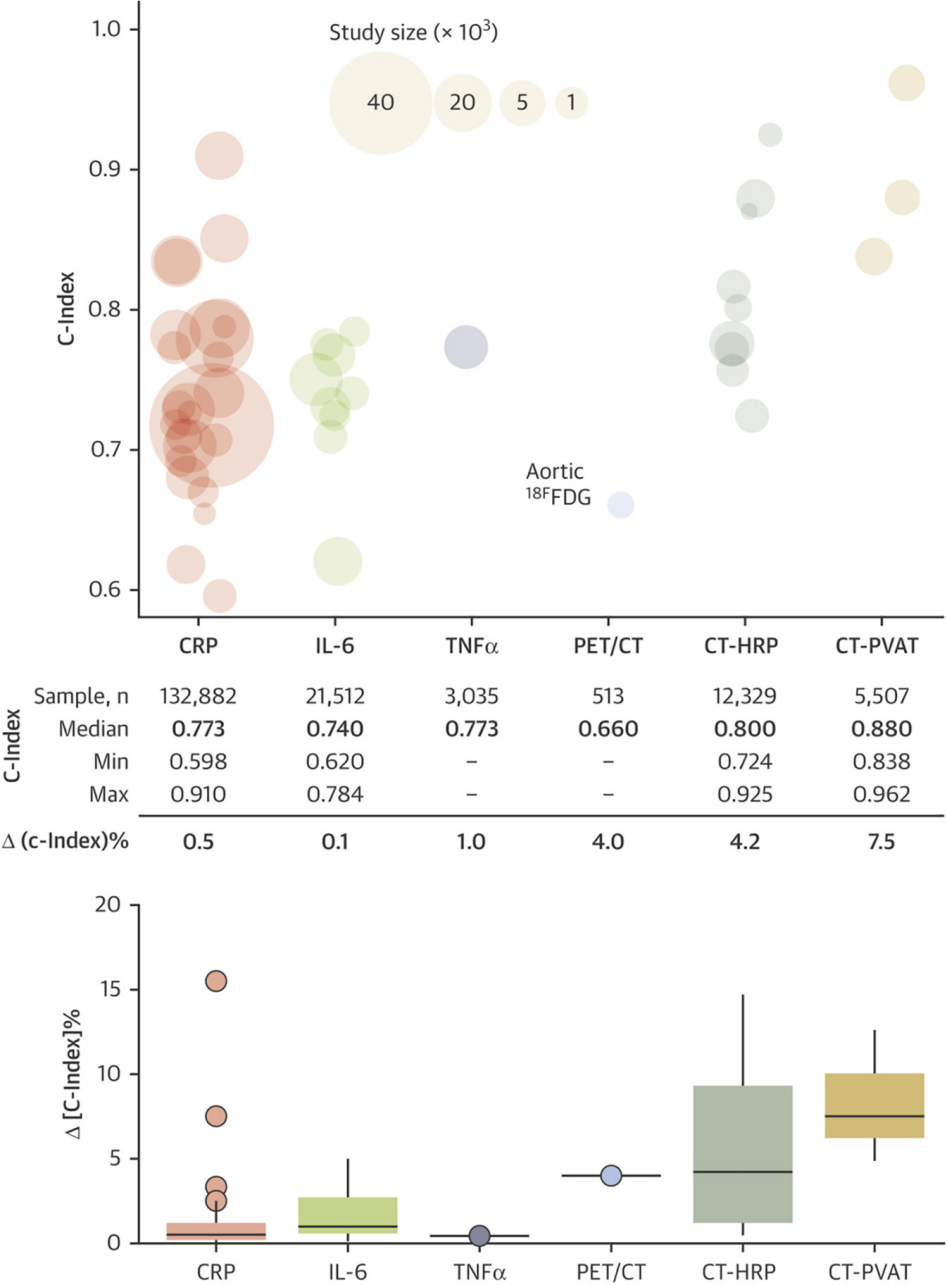
The Prognostic Performance (C-Index) of Inflammation Biomarkers for the Composite Endpoint of Major Adverse Clinical Outcomes and All-Cause Mortality Each “bubble” on the graph represents a study with its size being proportional to the sample size. Boxplots of the added prognostic value (Δ[c-index]) provided by each biomarker (calculated as the difference in C-index of the best clinical model in each study with the addition of the biomarker of interest; lower part of the panel). The added prognostic value of CCTA-HRP is reported in addition to coronary atherosclerosis extent, whereas that of CCTA-PVAT is reported in addition to coronary atherosclerosis extent and HRP. Reproduced with permission from Antonopoulos et al.^69^^18F^FDG = 18F-fluorodeoxyglucose; CRP = C-reactive protein; IL = interleukin; PET = arterial positron emission tomography; TNF = tumor necrosis factor; other abbreviations as in Figures 2, 3, and 4.

A relevant meta-analysis recently tested all current clinically available approaches to vascular inflammation detection and assessed their prognostic value.^69^ The measurement of vascular inflammation in addition to clinical risk factors was found to significantly enhance risk discrimination for cardiovascular events (Figure 5). PVAT assessment with CT imaging yielded the highest C-index values of all means assessed. Thus, coronary CTA biomarkers such as HRP features and pericoronary fat imaging through the FAI (alone or in combination) enhance cardiovascular risk discrimination beyond circulating biomarkers of inflammation.

### CT Radiotranscriptomics for the Detection of Vascular Inflammation

Handling CT scans as data sets for analysis rather than as images to examine with the human eye is the focus of the field of radiomics. Radiomics uses mathematical formulae to compute many hundreds of shape-, attenuation-, and texture-related features for a given anatomical volume or segmentation.^70^ Many radiomic features used together can be employed for disease diagnosis or prognostication. The field of radiomics was developed for the large-scale analysis of geospatial satellite imagery and first applied to health care in the field of cancer imaging.^71^ Radiomic approaches have now been implemented in coronary CTA with the aim of detecting biological mechanisms, including vascular inflammation occurring around the coronary arteries. Ultimately, the goal is to detect the residual inflammatory risk that continues to drive high morbidity and mortality associated with CAD.^72^

Using AI for radiotranscriptomic phenotyping of PVAT in risk prediction can potentially generate more advanced biomarkers for comprehensive PVAT phenotyping. This approach promises to unlock detection of precise subtypes of vascular pathology that determine texture and composition of PVAT through perivascular lipolysis, adipogenesis, edema, fibrosis, and angiogenesis. Basic science techniques such as RNA sequencing and histology serve to establish the biological “ground truth” of vascular pathology to which the radiomic signature of PVAT on coronary CTA is trained to specifically detect.

**Figure 6:**
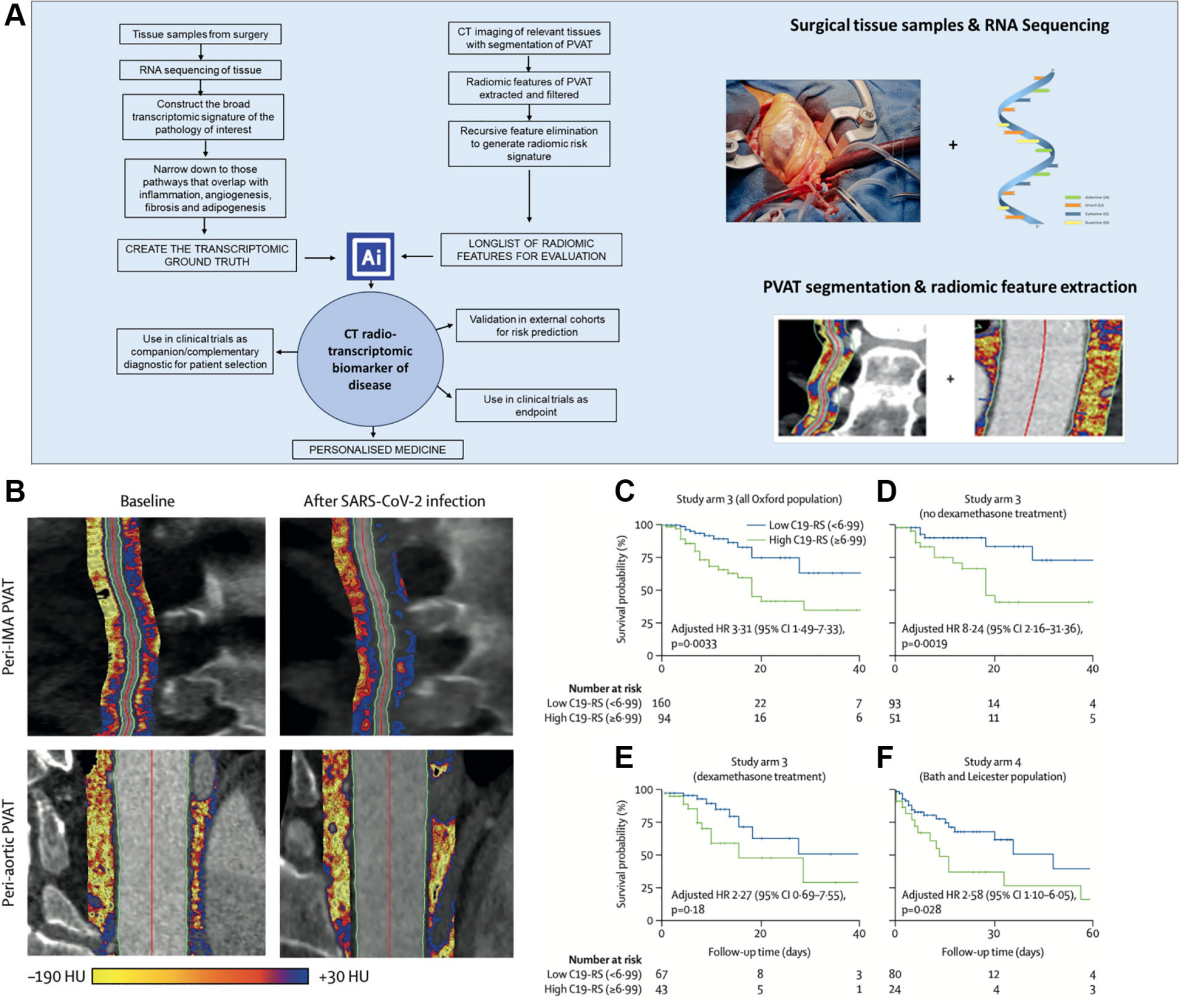
Radiotranscriptomic Detection of COVID-19 Severity Risk Using CT Detection of Perivascular Inflammation (A) Example workflow for the construction of CT radiotranscriptomoc biomarkers of vascular disease, with the merging of tissue markers such as RNA sequencing data with imaging detection of that disease processes via CT radiomics. (B) Illustration of perivascular adipose tissue mapping in CCTA in a patient 3 years before and during SARS-CoV-2 infection. (C) Kaplan-Meier curve and adjusted HR for in-hospital death for high vs low C19-RS groups in the SARS-CoV-2–positive study population (n = 254 [n = 139 from the first wave and n = 115 from the second wave]) with 39 deaths. HR adjusted for age >65 years, sex, cardiovascular risk factors (hypertension, hyperlipidemia, diabetes, body mass index, and presence of coronary artery disease), C-reactive protein plasma concentrations, white blood cell count, plasma troponin, history of chronic obstructive pulmonary disease, tube voltage, and dexamethasone treatment. (D) Kaplan-Meier curve and adjusted HR for in-hospital death for high vs low C19-RS groups in the SARS-CoV-2–positive study arm population who did not receive dexamethasone treatment (n = 144 with 19 deaths). (E) Kaplan-Meier curve and adjusted HR for in-hospital death for high vs low C19-RS groups in the SARS-CoV-2–positive study arm population who received dexamethasone treatment (n = 110 with 20 deaths). HRs in D and E adjusted for age >65 years, sex, cardiovascular risk factors (hypertension, hyperlipidemia, diabetes, and body mass index), C-reactive protein plasma concentrations, white blood cell count, history of chronic obstructive pulmonary disease, and tube voltage. (F) Kaplan-Meier curve and adjusted HR for in-hospital death for high vs low C19-RS groups in the external validation study arm 4 population (n = 104 with 34 deaths). HR adjusted for age >65 years, sex, cardiovascular risk factors (hypertension, hyperlipidemia, diabetes, body mass index, and presence of coronary artery disease), C-reactive protein plasma concentrations, white blood cell count, plasma troponin, history of chronic obstructive pulmonary disease, and tube voltage. Adapted with permission from Kotanidis et al.^76^ Abbreviations as in Figures 2, 4, and 5.

In cardiovascular imaging, the term “radiotranscriptomic” was introduced to describe this process of training radiomic signatures against the transcriptomic profile of the tissue. Figure 6A outlines the development pipeline for radiotranscriptomic imaging biomarkers. Two lines of work are needed: tissue samples from relevant PVAT with deep RNA sequencing and CT imaging of the relevant PVAT segments with full radiomic feature extraction. To limit analysis to radiomic features that have potential value as imaging biomarkers, filtering steps are undertaken to exclude the following: 1) features that are not stable in test–retest analyses; 2) features that are highly correlated with each other; and 3) features that are significantly correlated with other measures of adipose tissue volume. Importantly, recursive feature elimination with a random forest algorithm and repeated 5-times cross-validation have been shown to be a reproducible means to generate a final list of relevant radiomic features. Two examples of the development of radiotranscriptomic imaging biomarkers are outlined here, the fat radiomic profile (FRP) for CAD risk and the C19-RS for vascular inflammation attributable to COVID-19 infection.

In work that extended the use of CT radiomics beyond the power of a single radiomic feature for the assessment of changes in perivascular adipocytes that are reacting to vascular inflammation, we have reported the first study to apply complete radiomic feature quantification of coronary CTA scans for the purpose of better detection of vascular inflammation.^73^ Radiomic signatures derived via a machine learning approach were able to detect coronary artery inflammation, and features of radiomic texture were found to be related to adipose tissue fibrosis and vascularity, as measured through gene expression in tissue samples obtained during cardiac surgery. An AI-derived algorithm, the FRP, was applied to the SCOT-HEART trial, in which it significantly improved major adverse cardiac event prediction beyond traditional risk stratification that included risk factors, coronary calcium score, coronary stenosis, and HRP features on coronary CTA (Δ[C-statistic] = 0.126; *P* < 0.001).^74^ This represented an improvement in cardiovascular disease risk prediction beyond the current state of the art.

Notably, it was found that FRP was unlike perivascular FAI in relation to its responsiveness to therapy, as the FRP was not altered up to 6 months after an index cardiac event while FAI was shown to improve during this time; this suggests that FRP is detecting PVAT changes beyond coronary inflammation alone, changes that are not captured by FAI and that are less susceptible to current treatment strategies for CAD. These findings have been confirmed by other groups, including Lin et al,^75^ who recently showed in a prospective case-control study that patients with acute MI have a distinct PCAT radiomic phenotype compared with patients with stable or no CAD. These findings are important in understanding the mechanistic pathways that are being detected in these radiomic analyses, most importantly inflammation and fibrosis, and the possible translational applications of the technology into specific clinical scenarios related to the detection and monitoring of coronary artery inflammation.

In very recent work, we have also shown the role of CT radiomics for the detection of vascular inflammation and prognosis in patients with COVID-19 infection.^76^ This is another example of how radiotranscriptomics can be harnessed for vascular inflammation in a non-atherosclerosis setting. This technology will facilitate more effective trials of treatments and could identify patients at long-term risk of complications arising from their infection who may respond to therapy. An AI-powered radiomic “fingerprint” of the perivascular space (C19-RS), trained using as ground truth a transcriptomic signature of cytokine-driven inflammation (derived from RNA sequencing of human internal mammary arteries), had a striking prognostic value in conditions triggering acute vascular inflammation such as COVID-19.^76^ Technologies such as C19-RS are applicable to any type of contrast chest CT scan, gated (eg, CT coronary angiogram) or nongated (eg, CT angiogram of the pulmonary artery). It is important to note that C19-RS identifies an entirely distinct inflammatory phenotype from the pericoronary FAI and the FRP, with no overlap in radiomic features.

Indeed, patients with COVID-19 infection have much higher scores for C19-RS compared vs those without (Figure 6B). The score was found to have prognostic value for in-hospital mortality in COVID-19 in 2 testing cohorts (high [≥6.99] vs low [<6.99] C19-RS; HR: 3.31 [95% CI: 1.49-7.33; *P* = 0.0033] [Figure 6C] and HR: 2.58 [95% CI: 1.10-6.05; *P* = 0.028, respectively), adjusted for clinical factors, biochemical biomarkers of inflammation and myocardial injury, and technical parameters. C19-RS was also predictive of in-hospital death for high vs low C19-RS groups in the SARS-CoV-2–positive study arm population who did not receive dexamethasone treatment (Figure 6D) and those who did receive dexamethasone treatment (Figure 6E). HRs in Figures 6D and 6E are adjusted for age >65 years, sex, cardiovascular risk factors (hypertension, hyperlipidemia, diabetes, and body mass index), C-reactive protein plasma concentrations, white blood cell count, history of chronic obstructive pulmonary disease, and tube voltage. The risk was validated in an external sample from geographically distinct locations for in-hospital death for high vs low C19-RS groups (n = 104 with 34 deaths) (Figure 6F). Finally, C19-RS was strongly associated (*R* = 0.61; *P* = 0.00031) with a whole blood transcriptional module representing dysregulation of coagulation and platelet aggregation pathways. This radiotranscriptomic signature for in-hospital mortality in acute COVID-19 worked even when applied in non-gated CT angiograms of the pulmonary arteries.

Texture radiotranscriptomics can also be used to capture and quantify microcirculation in the perivascular space, in addition to lipolysis/adipogenesis, fibrosis, and edema, offering additional prognostic value over the perivascular FAI Score for cardiac events. Machine learning/radiotranscriptomic approaches are anticipated to revolutionize the utilization of adipose tissue as a tool for exploring vascular biology.

## Competing Technologies

### Ultrasound

Contrast-enhanced ultrasound has been investigated for visualization of inflammation in vessels affected by atherosclerosis. Although the techniques that have been developed have been useful in researching vascular inflammation, their clinical utility has not been fully realized. The potential future applications of ultrasound are particularly of interest as it could serve as a low-cost noninvasive modality for vascular inflammation detection.

The initial utility of ultrasound was in detection of early inflammation in the murine aorta, a classic animal model for cardiovascular disease. More recently, microbubbles targeted at VCAM type 1 (VCAM-1), a chemokine expressed by activated endothelial cells in atherosclerosis,^77^ have facilitated use of ultrasound scans to evaluate arterial inflammation initially in vitro.^78^ Noninvasive ultrasound has since been shown to detect VCAM-1 on human carotid arterial tissue using specialized microbubbles with a maleimide-thiol conjugation of an anti–VCAM-1 nanobody.^79^^,^^80^ Further in vitro research using contrast-enhanced ultrasound and von Willebrand factor A1–bearing microbubbles has been shown to detect activated platelets on vascular endothelium and indicate lesion severity in a rodent model of atherosclerosis.^81^ The use of microbubbles has not yet been validated in vivo in humans.

A recent translational study by Punjabi et al^82^ described the ability of VCAM-1–targeted microbubbles to detect treatment response to the glucagon-like peptide-1 agonist liraglutide by monitoring the VCAM-1 signal. This work shows the power of using biochemically targeted ultrasound imaging to prove molecular involvement in vivo. Intercellular adhesion molecule 1–targeted nano-ultrasonic contrast also has potential for future technological innovation to further our bottom-up understanding of atherosclerosis.^83^

Furthermore, echocardiographic molecular imaging of vascular endothelium has recently been shown to detect reductions in pro-inflammatory signals (eg, P-selectin, VCAM-1, von Willebrand factor) as a result of anti–interleukin-1β therapy.^84^ Ultrasound therefore has the potential to evaluate the effects of preclinically validated molecules on pro-atherosclerotic inflammation, which is critical to spurring future development of inflammation-targeted therapies.

Clinically, the utility of advanced ultrasound techniques for detecting carotid disease is being increasingly appreciated. Intraplaque neovascularization is a marker of carotid instability, a known precursor to embolic stroke, but is not detectable with Doppler ultrasound; contrast-enhanced ultrasound can detect it only invasively. Recently, however, superb microvascular imaging, a noninvasive technique that removes noise while preserving low-velocity flow signal, has been shown to accurately detect intraplaque neovascularization.^85^ A prospective cohort study comparing superb microvascular imaging against existing validated methods for detecting carotid plaque instability (eg, contrast-enhanced ultrasound, carotid-MRI and PET-^18^F-FDG, histology) is currently being conducted; the results have significant potential implications for detection of carotid inflammation and risk prediction of carotid ischemic events beyond extent of stenosis.^86^

Overall, although the results to date suggest that any ultrasound-based method of inflammation detection can be expected to have limited sensitivity relative to other imaging modalities, this is an area that could yield promising results in the future. This is especially true given that intravascular ultrasound has not yet been tested with the techniques discussed herein but could provide an exciting avenue for further research with greater resolution of the relevant tissues in real time.

### Magnetic Resonance Imaging

Along with echocardiography and PET, CMR imaging is widely considered a key tool for the diagnosis of inflammatory myocardial disease, with a Class I recommendation for the assessment of myocarditis and storage diseases in the current European Society of Cardiology guidelines for the diagnosis and treatment of acute and chronic heart failure.^87^ Despite CMR being shown to be an excellent noninvasive tool for the detection of myocardial inflammation in myocarditis, its utility in clinical detection of vascular inflammation has not been successfully achieved, as the means through which CMR detects myocardial disease do not map well to the pathobiology underlying the vascular inflammatory processes occurring in atherosclerosis. In detecting myocarditis, CMR does not directly assess immune cells but instead enables the assessment of macroscopic responses to inflammatory processes and myocardial injury at a low-resolution tissue level. The images acquired allow the identification of gross myocardial edema, vasodilation, fibrosis, and necrotic activity within the myocardium. Specific CMR sequences have been shown to be useful for the assessment of inflammatory pericardial disease. Such sequences include late gadolinium enhancement, T2 short tau inversion recovery, and viability phase-sensitive inversion recovery, which are also sequences that have been assessed for coronary artery inflammation detection.^88^

The use of non–PET-MR specifically for early detection of vascular inflammation is therefore very limited. Researchers continue to investigate means to allow the analysis of standard CMR acquisitions to be used for the identification of vascular inflammation as it would be an excellent addition to the clinical value achieved from this commonly performed noninvasive modality. Compared with CT imaging, CMR offers superior soft-tissue contrast, potentially allowing improved detection of HRP characteristics such as intraplaque hemorrhage, thrombus, and positive remodeling in large vessels. Importantly, CMR does not involve exposure to ionizing radiation; thus, if accurate means to assess both vascular plaque and the associated inflammation burden were discovered, they stand to be very powerful clinical tools.

### PET-CT Imaging

Molecular imaging technologies have emerged in the last decades as highly accurate techniques for the investigation of vascular inflammation in a noninvasive manner,^89^ allowing detection of clinically relevant atherosclerotic inflammation,^90^ with injectable tracers that bind pathophysiology-specific receptors. The tracers are then detected via PET. The exact mechanisms of the relevance of PET to cardiology have been thoroughly reviewed elsewhere.^91^ The most common intravenous tracer agent used in PET is ^18^F-FDG. This agent can noninvasively assess arterial inflammation as well as other inflammatory processes throughout the body. ^18^F-FDG uptake reflects glucose metabolism, which is particularly increased in inflamed atherosclerotic disease exhibiting the retention of macrophages and hypoxic stress.

^18^F-FDG has been used to assess the activity of metabolically active inflammatory cells and has shown a preference for inflamed coronary plaque in patients who have undergone proper preparation (low-carbohydrate, high-fat diet to suppress myocardial uptake of the tracer).^92^ Currently, ^18^F-FDG PET-CT imaging is used in the clinic to assess myocardial viability and blood flow, as well as to detect and monitor noncoronary conditions such as sarcoidosis and myocarditis. It is also well validated for the identification of carotid plaque instability.^85^

**Figure 7:**
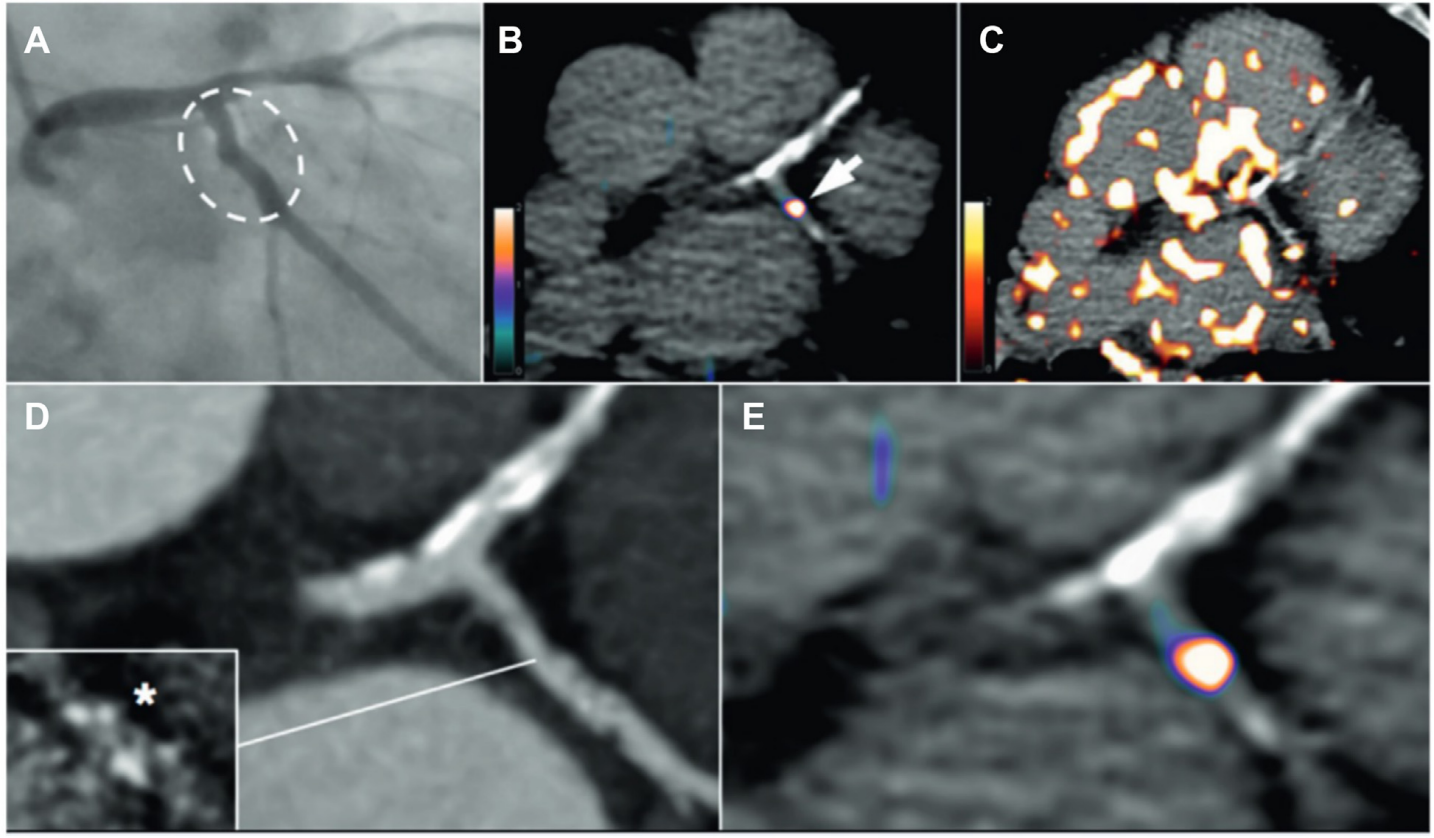
Coronary Artery PET Inflammation Imaging With 68Ga-DOTATATE Radiograph (A) and CT coronary angiograms (D) of a 67-year-old man with stable angina, showing minor left circumflex atheroma (hatched oval) with spotty calcification ([inset] ∗calcium scan) and calcified plaque in the left anterior descending artery. Although gallium-68-labeled DOTATATE (^68^Ga-DOTATATE) positron emission tomography (PET) (B, E) allows unimpeded interpretation of inflammation in the left circumflex artery lesion (B, arrow), and lack of signal in the left anterior descending artery, coronary ^18^F-fluorodeoxyglucose imaging is obscured by patchy myocardial tracer uptake (C). Adapted with permission from Tarkin et al.^93^

Another radiotracer, gallium-68-labeled DOTATATE (^68^Ga-DOTATATE), can also provide clinically useful images of vascular inflammation. This tracer is widely used in imaging neuroendocrine tumors and binds to the somatostatin receptor subtype 2 (SST_2_), which is expressed by M1 pro-inflammatory macrophages. PET-CT images using ^68^Ga-DOTATATE have been found to produce better image quality than ^18^F-FDG and can be used to identify inflamed coronary lesions^93^ and residual inflammation in myocardial tissue after an acute MI.^94^ Figure 7 illustrates the use of ^68^Ga-DOTATATE for detection of coronary artery vascular inflammation compared with ^18^F-FDG.

Several other radiotracers have also shown promise in imaging inflammation within the coronary wall. For instance, ^18^F-NaF, which has a strong affinity for the vascular wall, has been shown to incorporate into hydroxyapatite in areas of arterial wall microcalcification. Imaging with ^18^F-NaF has shown promising accuracy in detecting the culprit coronary lesions and abdominal aortic aneurysms,^95^ with ^18^F-NaF PET-CT imaging being the first noninvasive imaging method to identify and localize ruptured and high-risk coronary plaque.^96^ ^18^F-NaF has been used to investigate developing microcalcification in the vasculature. Work from Dweck et al^97^ showed that coronary uptake was associated with cardiovascular risk, describing significant associations between coronary arterial NaF uptake and prior coronary events, angina status, and Framingham risk scores. Recent work from the same group has shown that in a small cohort of patients with established CAD pooled from a prospective observational study, ^18^F-NaF PET provides powerful independent prediction of fatal or nonfatal MI.^98^ ^18^F-fluciclatide, a PET tracer that binds to α_v_β_3_ integrin, is also considered a promising tool for identifying high-risk coronary plaque.^99^ Indeed, small studies in humans have shown that the quantification of α_v_β_3_ integrin expression with ^18^F-fluciclatide PET has potential to assess plaque vulnerability and disease activity in aortic atherosclerosis.^100^

There is early evidence to suggest that the 18 kDa translocator protein could be used as a target for imaging inflammation using a specific PET-CT tracer. For example, ^11^C-PK11195 has been used to this end, although primarily in carotid disease cases. PET-CT imaging using ^11^C-PK11195 has been found to distinguish between recently symptomatic and asymptomatic plaque in carotid disease populations.^101^^,^^102^

Other experimental techniques have also been explored to visualize coronary artery inflammation using PET-CT imaging, such as visualizing chemokine receptors, which are up-regulated in pro-inflammatory macrophages, in experimental nanoplatforms,^103^ or visualizing endothelial activation and inflammation using ^18^F-labeled small VCAM-1 affinity ligands.^104^ Magnetic resonance and optical detection technologies can be combined with PET to visualize inflammation by using ^64^Cu-labeled 20 nm magnetofluorescent polysaccharide-containing nanoparticles,^105^ which have been found to accumulate in macrophages in atherosclerotic lesions in apolipoprotein E knock-out mice.

Single-photon emission CT imaging/CT imaging that uses ^111^In- and ^123^I-radiolabeled compounds is another experimental approach to noninvasively visualize coronary artery inflammation. These compounds target activated matrix metallopeptidases and allow the detection and tracking of plaque composition in response to treatment.^106^

Although ^18^F-FDG PET-CT imaging and increasingly ^18^F-NaF are widely used for myocardial assessment, the other mentioned techniques are currently experimental and restricted by their high costs, limited availability, and required expertise. As we have discussed, ^18^F-FDG accumulation occurs with PET-CT imaging in vulnerable atherosclerotic plaque. From a clinician's perspective, the main obstacle to wider adoption of these techniques is a lack of clinical availability, lack of expertise, and, most importantly, a paucity of clinical evidence to show the translational potential of these PET-CT techniques for reduction of either primary or secondary cardiovascular risk.

### PET-MRI Technology

As discussed earlier in relation to PET-CT imaging, ^18^F-FDG can assess arterial inflammation in vivo because tracer uptake reflects, at least in part, the presence of invading inflammatory cells, including macrophages and foam cell formation.^107^ MRI can also provide atherosclerotic plaque detection and characterization; thus, hybrid PET-MRI technology has the potential to simultaneously provide anatomical and functional information.^108^ However, data on vascular PET-MRI remain scarce, with the focus of research on carotid atherosclerosis.^109^

In recent work focused on the detection of vascular inflammation in patients with large vessel vasculitis, it has been shown that PET-MRI can be used to detect active inflammation via the detection of SST_2_ as a novel inflammation-specific molecular marker.^110^ SST_2_ is expressed by inflammatory macrophages activated in vitro, and SST_2_ staining colocalizes with CD68^+^ macrophages within inflamed atherosclerotic plaques. Of the somatostatin receptor PET tracers used for clinical neuroendocrine tumor imaging, ^68^Ga-DOTATATE has the highest binding affinity for SST_2_. A novel ^18^F click-labeled octreotide radioligand called ^18^F-FET-βAG-TOCA has also shown high SST_2_-binding affinity and favorable tracer kinetics. SST_2_ PET-MRI was found to be consistent with ^18^F-FDG PET-CT imaging in patients with large vessel vasculitis. Resolution of the PET-MRI clinical images was found to be suitable for coronary and intracranial arteries.

Other experimental PET-MRI probes are currently in development for inflammatory purposes. These include ^18^fluorine-fluoromethylcholine, which has shown better identification of atherosclerotic plaque and lower myocardial uptake compared with ^18^F-FDG in murine models. Current trials of this probe include a PET-MRI study recruiting those with ACS who will undergo optical coherence tomography to investigate if intravascular findings of high-risk plaque correlate with ^18^fluorine-fluoromethylcholine uptake on PET-MRI (NCT03252990). There are also a number of small clinical studies investigating the use of cell adhesion motifs for inflammatory imaging in atherosclerosis. One of the most advanced is tripeptide Arg-Gly-Asp (RGD), which was originally identified as the sequence within fibronectin that mediates cell attachment. The RGD motif has now been found in numerous other proteins and has been identified as a key molecule that supports cell adhesion within atherosclerotic plaque. An ongoing ^18^F-FPPRGD2 study is exploring the use of these probes in both PET-MRI and PET-CT imaging in a cohort of carotid endarterectomy patients (NCT02995642).

## Conclusions

The field of noninvasive clinical imaging of vascular inflammation has undergone significant advancements in recent years (Table 1). The integration of various imaging modalities into clinical practice such as CT and molecular imaging with PET-CT imaging has allowed for a more comprehensive and accurate assessment of the presence and extent of vascular inflammation, particularly in the coronary arteries. The application of image analysis techniques on imaging data sets (eg, perivascular FAI Score and radiotranscriptomic phenotyping from routine coronary CTA) have shown very promising results in the use of vascular inflammation as part of CAD risk stratification. Ongoing studies are expected to validate their clinical utility, economic feasibility, and overall impact on clinical management in primary and secondary prevention.

**Table 1:** Detailed Summary of Imaging Modalities for Detection of Vascular Inflammation

	USS	Cardiac MRI	CT Imaging	PET-CT Imaging	PET-MRI
					
Traditional usage^a^	Assessment of heart function, including movement of myocardium and valves	Assessment of structural disease processes cardiomyopathies and pericardial disease, and myocardial viability	Assessment of coronary artery disease and congenital heart disease	Detection of ischemic and nonischemic cardiomyopathy and cardiac tumors	Not currently in widespread usage
Vascular inflammation usage	Accurate detection in murine models Nanobubbles + USS may indicate lesion severity in vitro	None validated at present	Detection of vascular inflammation via the perivascular fat attenuation index, with proposed diagnostic and prognostic usage	Detection of inflamed coronaries and residual post-MI inflammation Allows for tracking of plaque composition	Can detect active inflammation via SST_2_
Strengths	Low cost Truly noninvasive Ease of use IVUS may revolutionize	Potential for approach to vascular inflammation similar to that of myocardial inflammation	Rapid examination Validated tools for coronary artery inflammation detection	Molecular imaging with novel tracers allow targeted detection of inflammation; gold standard for tissue inflammation detection	Use of tracers allows for tissue-level resolution Resolution without radiation
Limitations	Low- resolution and no 3D analysis Clinical evidence lacking Unable to assess adipose around vessels	Low tissue-level resolution High cost Vascular assessment of large vessels only	Radiation exposure IV contrast needed	Requires access to advanced imaging center Poor spatial resolution	Lack of clinical availability and expertise Paucity of clinical evidence Cannot image adipose tissue

3D = 3-dimensional; CT = computed imaging; IV = intravenous; IVUS = intravascular ultrasound; MI = myocardial infarction; MRI = magnetic resonance imaging; PET= positron emission tomography; SST2 = somatostatin receptor 2; USS = ultrasound scan.

a Traditional usage in brief; full indications are broader.

## Funding Support and Author Disclosures

Dr Antoniades declares several patents (US10,695,023B2, PCT/GB2017/053262, GB2018/1818049.7, GR20180100490, and GR20180100510) licensed to Caristo Diagnostics. Dr Antoniades is the Chair of the British Atherosclerosis Society, as well as founder, shareholder, and director of Caristo Diagnostics, a University of Oxford spinout company. He declares past honoraria from Amarin, Silence Therapeutics, and Caristo Diagnostics; and funding from the British Heart Foundation (CH/F/21/90009 and RG/F/21/110040), the British National Institute for Health and Care Research, and the Oxford Biomedical Research Centre. All other authors have reported that they have no relationships relevant to the contents of this paper to disclose.
